# Multiple rare organisms causing ventriculoperitoneal shunt infection and brain abscess

**DOI:** 10.5339/qmj.2023.7

**Published:** 2023-02-20

**Authors:** Omar M. Shihadeh, Hayel Amin, Firas Hammadi

**Affiliations:** ^1^Hamad Medical Corporation, Hamad General Hospital, Neurosurgery Department. E-mail: oshihadeh@hamad.qa ORCID ID: 0000-0001-8505-5147

**Keywords:** Ventriculoperitoneal shunt Infection meningitis Abscess Neurosurgery Complications

## Abstract

Ventriculoperitoneal shunts (VPS) are inserted to treat issues with CSF flow dynamics, such as obstructions causing CSF build up which raises the intracranial pressure. A major complication of this procedure is VPS infections. Vast majority of VPS infections are monomicrobial and may occur in the first two years of insertion due to contiguous or hematogenous spread. Here, we report a rare case of polymicrobial VPS infection with 5 pathogens. One of these organisms (*Citrobacter werkmanii*) has been reported as a cause of meningitis for the first time in this report. The other organism (*Enterococcus casseliflavus*) has been reported as a cause only one other time. Hence, we recommend considering these newly emerging organisms when dealing with meningitis.

## Introduction

Polymicrobial ventriculoperitoneal shunt (VPS) infections are caused by two or more pathogenic microorganisms. Lia et al. determined^1^ that the incidence of VPS is 14% and accounts for 2.3% of all cases of meningitis. The most commonly implicated pathogen in polymicrobial meningitis are gram-negative strains (76%), followed by gram-positive strains (24%).^2^

The vast majority of reported cases of polymicrobial infections often describe only 2–3 causative pathogens; occasionally, four or more pathogens have been described.^2-4^

*Citrobacter* species are considered among the rarest causes of meningitis, specifically affecting neonates and infants. The most commonly implicated species are *citrobacter koseri* and *citrobacter diversus*.^5^ Our patient's culture showed *citrobacter werkmanii* growth which is extremely rare. To the best of our knowledge, this is the first report of *citrobacter werkmanii* as the cause of meningitis in literature, specifically for VPS-associated meningitis.

*Enterococci* species account for only 5% of all VPS-associated meningitis, and are predominantly caused by either *Enterococcus faecalis* or *Enterococcus faecium*. This case report is only the second in literature to report *Enterococcus casseliflavus* as the causative pathogen for VPS infection.^6^

## Case Presentation

A 33-year-old male patient with a history of VPS insertion in 2007 for congenital malformation-induced hydrocephalus of unknown etiology, presented to the hospital. His VPS was revised due to an infection in 2019 at an outside facility. He did not experience any other VPS complications (e.g., mechanical malfunction, misalignment, or blockade) during this time period. He presented to the emergency department of our hospital with complaints of headache, nausea, and three episodes of vomiting of one-day duration, with no flu-like symptoms or contacts with a sick person. A review of each organ system was unremarkable. Physical examination revealed all normal vital signs except for a fever (38.0 °C). The patient has mild neck stiffness but no papilledema, photophobia, or signs of meningeal irritation.

Cerebrospinal fluid (CSF) analysis revealed a white blood cell (WBC) count of 3,778 /uL, with 57% neutrophils and 42% lymphocytes (normal WBC count < 5, without neutrophils), and CRP levels of 39 mg/L. A complete blood cell count revealed a WBC count of 11.4x10^3/uL and creatinine levels of 1.4 mg/dl. Other laboratory tests were normal.

**Figure 1.**
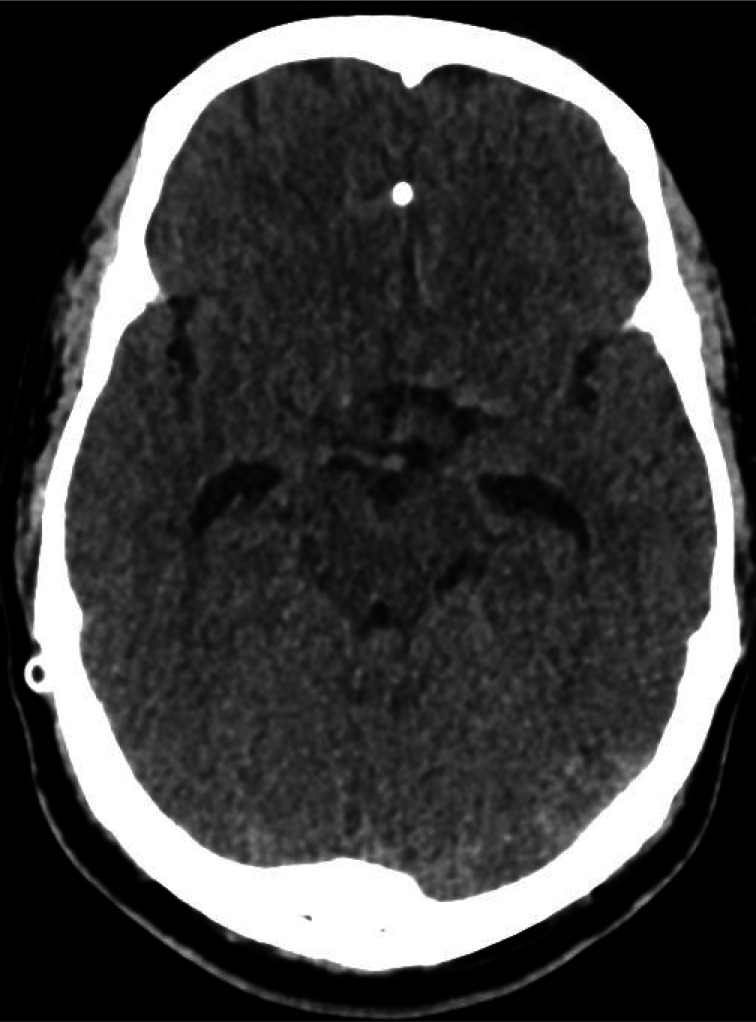
Tip of the VPS is visualized lying outside the lateral ventricle.

**Figure 2.**
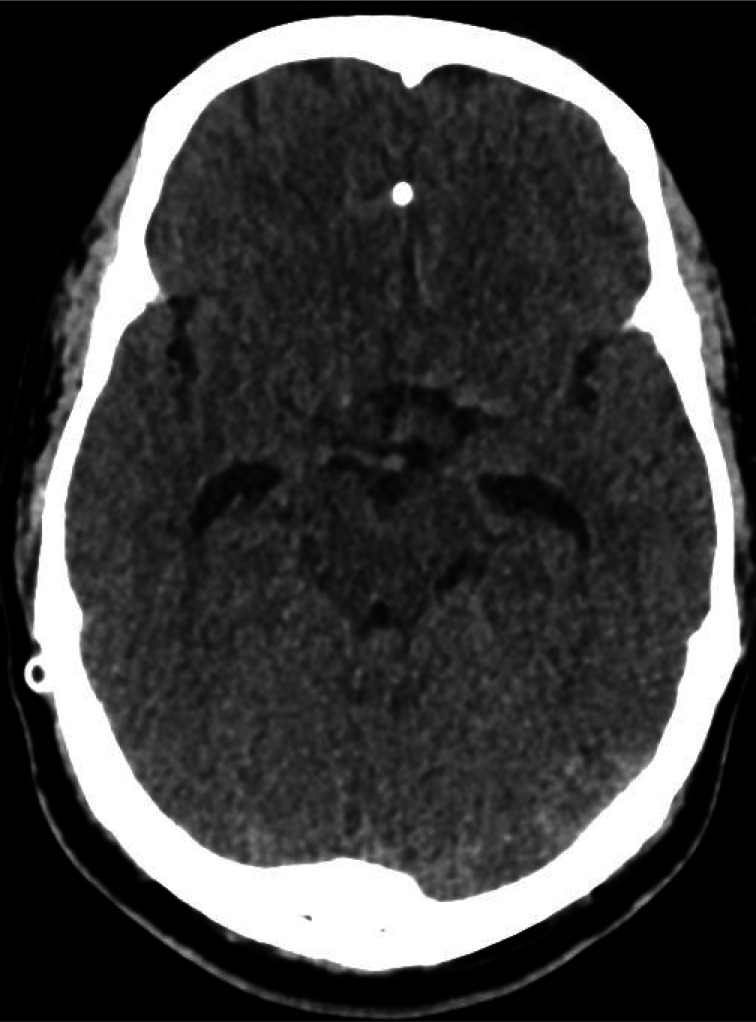
The temporal horns are dilated which indicate hydrocephalus.

Computed tomography (CT) of the head showed a right-sided VPS with the tip outside the lateral ventricle, mild hydrocephalus, and a midline shift of 4 mm (Figures 1 & 2).

The VPS was removed, and an external ventricular drain (EVD) was inserted. Only the distal end of the shunt could be removed. The proximal end was firmly adherent and was, thus, tied with silk sutures and retained. The patient was admitted to the ICU for two days and then shifted to the neurosurgery ward. A postoperative brain CT showed resolution of the hydrocephalus and the midline shift.

CSF cultures obtained intraoperatively from within the VPS before removal yielded only *Enterococcus casseliflavus*. Cultures of the VPS tip grew five different microorganisms: *Enterococcus casseliflavus*, *Escherichia coli*, *Citrobacter werkmanii, Enterobacter cloacae*, and *Klebsiella oxytoca*. The patient was started on intravenous ampicillin and cefepime depending on the antibiotic sensitivity of each organism (Table 1).

**Figure 3.**
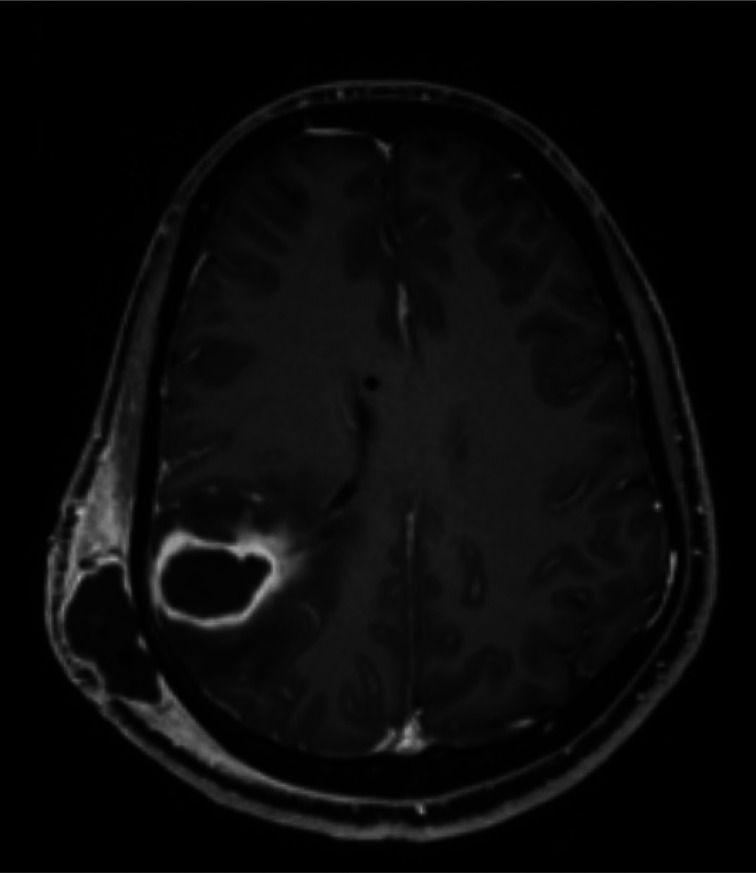
Contrast MRI showing a ring-enhanced lesion within the previous VPS pathway and connected to the subcutaneous tissue

Two weeks after surgery, a brain MRI showed a right frontoparietal abscess communicating with an extraaxial/scalp collection at the site of the previous burr hole and the VPS tract (Figure 3). The following day the patient underwent craniotomy for abscess evacuation and EVD revision. He was retained in the ICU for three days, following which he was transferred to the ward. A postoperative brain MRI showed complete evacuation of the intracranial abscess.

The abscess culture yielded *Citrobacter amalonaticus* and *Enterobacter cloacae.* The antibiotic regimen was changed to intravenous ampicillin, linezolid, and meropenem, according to antibiotic sensitivity (Table 2).

Ten days after abscess evacuation, MRI of the brain showed an increase in the size of the and extracranial collections at the surgical site with a connection to the right frontoparietal cavity. The patient underwent wound exploration, removal of the infected bone, and EVD revision.

Six weeks following the second abscess evacuation, the patient improved clinically, radiologically, and biochemically, including the CSF parameters. He underwent right EVD removal and left VPS insertion. He was discharged after completion of the antibiotic regimen.

The total hospital stay duration was 55 days, the last follow-up was in the clinic 112 days after the initial presentation (57 days after the discharge), where he was seen by the operating consultant neurosurgeon, and no complaints were recorded, and examination was within normal limits. The patient was scheduled for brain MRI and subsequent implant cranioplasty, but unfortunately, he left to his home country. We tried to reach out to him, but we could not.

## Discussion

VPS is one of the commonest procedures in neurosurgical practice. A significant problem encountered in this procedure is an infection, with an incident rate ranging from 2 to 27% and often resulting in a poor outcome.^7^ According to the largest cohort study conducted by Pelegrin et al., most cases of VPS infections are monomicrobial (86%) and are predominantly caused by *Staphylococci* (65%). Gram-negative bacteria are responsible for approximately 15% of the cases and are predominantly caused by the *Enterobacteriaceae* species.^1^ Polymicrobial infections of VPS are not uncommon (14%). The vast majority of these mixed infections are caused by 2–3 pathogens, and, very rarely, by four or more.^2,3,8^

Our patient had a history of VPS revision due to an infection 12 years after the primary surgery. This is in accordance with the findings of Abuhadi et al.,^9^ who demonstrated a higher risk of VPS infection associated with infection-associated shunt reinsertions. Our patient developed another VPS infection one year after the revision surgery. This case report demonstrated the rare finding of VPS infection being caused by five different microorganisms. Additionally, a different species was isolated from the subsequent brain abscess. Thus, a total of six different species were isolated from the same patient, of which two have not been reported to date.

*Citrobacter werkmanii* is a rare Gram-negative enteric bacillus that belongs to the *Enterobacteriaceae*, and is found in the intestinal tract of humans and animals and in soil and water. It primarily causes urinary tract infections.^10^ Cases of Citrobacter species-associated meningitis are reportedly very rare in adults; most cases have been reported in neonates and are predominantly caused by *Citrobacter koseri* or *Citrobacter diversus*.^11^ To the best of our knowledge, *Citrobacter werkmanii* has never been reported to be the cause for VPS-associated (nor other CNS implant-associated) meningitis in literature.

Local contamination of VPS from the scalp skin cannot be entirely ruled out. However, as *citrobacter* is not a normal skin flora, it is relatively certain that the skin not the source of contamination. As *citrobacter* can be isolated from intestinal tract, ascending infection may be a possibility via the distal end of the VPS which is placed intra-peritoneally.

*Enterococcus casseliflavus* has only been reported once as a cause of meningitis by Iaria et al. Enterococcal meningitis is an uncommon disease accounting for only 0.3–4% of cases of bacterial meningitis which is associated with a high mortality rate. *Enterococcus faecalis* and *Enterococcus faecium* are the only two species that have reported to cause meningitis in patients.^2^

Three of the isolated organisms belong to the *Enterobacteriaceae* genus, primarily: *Enterobacter cloacae*, *Klebsiella oxytoca*, and *Citrobacter werkmanii*. The other two isolated microbes were *Escherichia coli*, which is a gram-negative, facultative anaerobic bacteria responsible for 2% of VPS meningitis, and *Enterococcus casseliflavus*. As reported by Pelegrin et al., *Enterobacter cloacae* and *Klebsiella oxytoca* are responsible for 1% of the cases and *Escherichia coli* are responsible for 2% of the cases. The other two organisms have not been reported till date.

The microorganism responsible for the late development of abscess in this patient was *Citrobacter amalonaticus*, which is associated with 75% of cases with abscess formation.^5^ The source of this bacteria could not be ascertained. However, it is highly unlikely to have been introduced, even accidentally, as the abscess was located in the tract of the removed VPS and EVD. Furthermore, the new VPS and EVD were introduced through another tract. Thus, this pathogen may have already been present during the original polymicrobial meningitis.

The antibiotic sensitivity for these pathogens showed no multidrug resistance, and the patient was initiated on empirical ceftriaxone and vancomycin, followed by meropenem (for ESBL organisms), linezolid, and ampicillin (for enterococcus casseliflavus). All antibiotics were administered intravenously without the need for intrathecal administration.

*Enterococcus casseliflavus* can be vancomycin-resistant due to its inherited genome, which is prominent in only some enterococci stains, such as *E. gallinarum*.^12^

It is difficult to rank these organisms according to pathogenicity as each of them present with different pathogenic features and are associated with several other serious infections.

CSF culture (through lumbar puncture) is a minimally invasive method for detecting bacterial meningitis, with 70% sensitivity^13^; however, in case of the presence of an implant, the implant should be removed and sent for culture. The implant could be a seeding source of pathogens, especially of those capable of forming biofilms (e.g., E. coli in this patient).^14^ In our case CSF culture yielded only one organism in comparison with the five organisms yielded via VPS tip culture. Hence, depending on CSF culture alone would have resulted in inadequate treatment.

**N.B.** ESBL organisms: Escherichia coli and Klebsiella pneumoniae, VPS: ventriculoperitoneal shunt

## Conclusion

We have reported a rare case of polymicrobial meningitis complicated by brain abscess, which had been caused by six different rare organisms, including *Citrobacter werkmanii*, which has not been mentioned in literature yet, and *Enterococcus casseliflavus*, which has been reported only once. We recommend considering these newly emerging organisms when dealing with meningitis and the multiple antibacterial regimens to be instilled.

## Figures and Tables

**Table 1.**
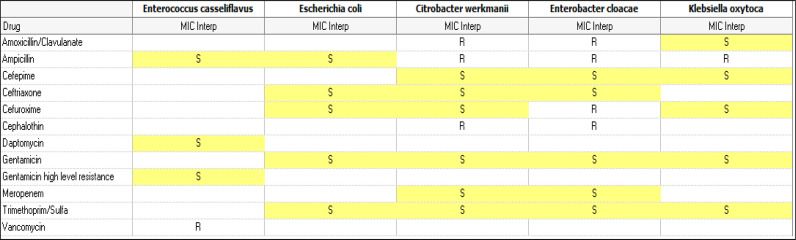
The antibiotic sensitivity for each organism isolated from the VPS tip culture.

**Table 2.**
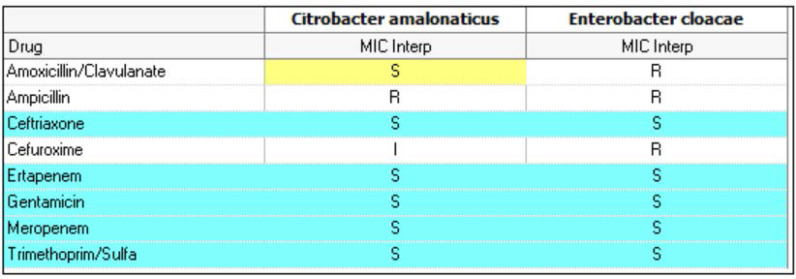
The antibiotic sensitivity for both bacteria isolated from the abscess culture.
